# CircFMN2 Boosts Sorafenib Resistance in Hepatocellular Carcinoma Cells via Upregulating CNBP by Restraining Ubiquitination

**DOI:** 10.1155/2022/2674163

**Published:** 2022-07-21

**Authors:** Chen Fan, Xiaoli Zhu, Qi Zhou, Weidong Wang

**Affiliations:** ^1^Department of Intervention, Wuxi People's Hospital, Nanjing Medical University, Wuxi 214023, China; ^2^Department of Intervention, The First Affiliated Hospital of Soochow University, Suzhou 215006, China

## Abstract

**Purpose:**

Noncoding RNAs exert critical biological effects in hepatocellular carcinoma (HCC). The role of circFMN2, a newly discovered functional RNA in prostate cancer and colorectal cancer, was investigated for the first time in sorafenib-resistance HCC cells.

**Methods:**

The level of circFMN2 was assessed via quantitative real-time PCR (qRT-PCR). Cell proliferation was detected via CCK-8 and colony formation assay. Cell apoptosis was measured via the TUNEL assay and flow cytometry analysis. A Western blot assay was conducted to detect the CCHC-type zinc finger nucleic acid binding protein (CNBP) level and ubiquitination. RNA pull-down assay and RNA immunoprecipitation were carried out to explore the interaction between circFMN2 and CNBP.

**Results:**

CircFMN2 was highly expressed in multidrug-resistant (MDR) cells. CircFMN2 overexpression exerted pro-proliferation effects in sorafenib-treated HCC cells, while depletion of circFMN2 displayed negative effect on sorafenib-treated MDR cells. Moreover, CNBP was verified as the binding protein of circFMN2. CNBP was upregulated in MDR cells, which was achieved by inhibition of ubiquitination by circFMN2. Besides, CNBP overexpression was found to boost sorafenib resistance in HCC cells.

**Conclusions:**

CircFMN2 is aberrantly expressed in sorafenib-resistant HCC cells and contributes to sorafenib resistance in HCC cells via upregulation of CNBP by restraining ubiquitination.

## 1. Introduction

Hepatocellular carcinoma (HCC) has been a tremendous health and economic burden globally. Among all kinds of cancers, liver cancer, or HCC, is the second most fatal cancer with a death rate of over 90% and its prevalence is still trending up worldwide [1, 2]. It was disclosed by the World Health Organization in the annual projections that deaths from HCC will be over one million in 2030 [3]. A growing concern is needed for the prevalence of hepatic carcinoma. Although considerable progress has been achieved in the treatment of hepatic carcinoma such as microwave ablation, radiofrequency, liver resection, chemotherapy, and liver transplantation, there are still many intractable obstacles including low diagnosis rate, high postoperative recurrence, drug resistance, and poor survival rates [4–11]. There is a great need to find the effective therapeutic targets, risk factors for drug resistance, and efficient diagnostic markers.

Due to the inconspicuous symptoms at an early stage, HCC cases are often confirmed at advanced stages, missing the opportunity of surgical treatment or ablation. Hence, a systematic targeted therapy has raised considerable interest. Sorafenib is a first-line FDA-approved systematic targeted therapeutic drug, exerting crucial therapeutically effects on HCC at later stages [12, 13]. In clinical practice, its benefit in survival after sorafenib therapy has been fully validated [14–16]. However, considering the prevalence of HCC, therapeutic breakthroughs on sorafenib resistance and existing treatment efficiency are still concerned.

CircRNAs with a covalently closed circular structure are a class of stable functional molecules and have been confirmed as vital regulators in the diagnosis, treatment, and drug resistance in HCC. Circ_100395 exerts anticancer effects in HCC via regulating epithelial-mesenchymal transition, apoptosis, and proliferation [17]. Circ_0003418 improves cisplatin chemoresistance via suppression of Wnt/*β*-catenin pathway in HCC [18]. CircUHRF1 contributes to anti-PD1 therapy resistance via disturbing NK cell function in HCC [19]. CircFoxo3 drives adriamycin resistance via modulating the miR-199a-5p/ABCC1 axis in HCC [20]. The effects of most circRNAs in HCC remain unknown and so far, there are few reports on the circRNAs regulating sorafenib resistance. CircFMN2 is a newly discovered circRNA involved in prostate cancer and colorectal cancer [21, 22], and its functions in sorafenib resistance in HCC remains undefined. CCHC-type zinc finger nucleic acid binding protein (CNBP, also known as ZNF9) is a conserved single-stranded DNA binding protein, which has been shown to participate in the metabolism of HCC cells [23], but if it helps or prohibits the development and growth of HCC is still unclear. In this study, we explored the role of circFMN2 in sorafenib resistance and its underlying mechanism in HCC.

## 2. Methods

### 2.1. Cell Culture and Treatment

HCC cell (BEL-7402) and multidrug-resistant HCC cell (BEL-7402/5-Fu) were purchased from Wuhan Chundo Biotechnology Co. LTD. (Wuhan, China). The BEL-7402 and BEL-7402/5-Fu cells were cultured in Dulbecco's modified eagle medium (DMEM) (Gibico, Rockville, MD, USA) containing 100 *μ*g/ml streptomycin, 100 IU/ml penicillin, and 10% fetal bovine serum (FBS) (Gibico, Rockville, MD, USA) at 37°C in an incubator with 5% CO_2_. For the role of circFMN2 and CNBP in sorafenib (SOR) resistance, the cells were treated with sorafenib (6.5 *μ*mol/L) for 24 h.

### 2.2. Cell Transfection

After sorafenib treatment, the BEL-7402 cells were transfected with pcDNA3.1-CircFMN2 vector, pcDNA3.1-CNBP vector, and their corresponding negative controls (BlueGene Biotech, Shanghai, China). BEL-7402/5-Fu cells were transfected with siRNA and its negative control (si-circFMN2 5′-AAGAAAGACTTGAAAGCTGTT-3′; si-circFMN2-NC 5′-GUGAGGCUCUUGAGCCAGAUGAUTG-3′; BlueGene Biotech, Shanghai, China) using Lipofectamine 3000 (Invitrogen, Carlsbad, CA, USA) on the basis of manufacturer's instructions.

### 2.3. Quantitative Real-Time Polymerase Chain Reaction (qRT-PCR)

Isolation of total RNAs was conducted using RNAprep Pure cell kit (TianGen Biotech, Beijing, China). HiFiScript complementary deoxyribose nucleic acid (cDNA) Kit (CWBIO, Beijing, China) was used to synthesis cDNA. CircFMN2 primers (forward: 5′- TCAGAAACTCCCCAAAAACG-3′, reverse: 5′-AGAAGACCCATGGCAATGAT-3′) and other primers were synthesized by BlueGene Biotech, Shanghai, China. Quantitative analysis was carried out in triplicates on StepOne Plus Real-time PCR System (Applied Biosystems, Foster City, CA, USA) using SYBR Green. U6 (forward: 5′-GCTTCGGCAGCACATATACTAAAAT-3′, reverse: 5′-CGCTTCACGAATTTGCGTGTCAT-3′) served as an internal reference. The quantitative calculation was done using 2^−∆∆Ct^ methods. Experiments were triplicated.

### 2.4. Cell Counting Kit-8 (CCK-8) Assay

The cell viability was measured using CCK-8 (GlpBio, Shanghai, China) in line with the instructions of manufacturer. Briefly, 2 × 10^3^ cells were seeded into each well of the 96-well plate and after sorafenib treatment and transfection, cells in each well were incubated with CCK-8 solution (10 *μ*L) for 2 h. The cell viability was determined by measuring the absorbance at 450 nm. Experiments were triplicated.

### 2.5. Colony Formation Assay

The cells after sorafenib treatment and transfection were seeded in six-well plates. Fourteen days later, 4% paraformaldehyde fixation was carried out followed by crystal violet staining. The colonies were counted and observed under a microscope (Olympus, Tokyo, Japan). Experiments were triplicated.

### 2.6. Flow Cytometry Assay

The cell apoptosis was evaluated using Annexin V-FITC/PI Apoptosis Detection Kit (YEASEN, Shanghai, China) as described in the instructions of the manufacturer. Briefly, the cells after sorafenib treatment and transfection were digested with trypsin followed by centrifugation at 4°C; the cells were resuspended in the binding buffer (100 *μ*L). Then, 5 *μ*L Annexin V-FITC and 10 *μ*L PI staining solution were incubated with the cells away from light at room temperature for fifteen minutes. The analysis of cell apoptosis was carried out using the FACScan flow cytometer (Becton Dickinson, Franklin Lakes, NJ, USA). Experiments were triplicated.

### 2.7. TUNEL Assay

The cells apoptosis was measured using Colorimetric TUNEL Apoptosis Assay Kit (Beyotime, Shanghai, China) in accordance with the instructions of the manufacturer. Briefly, the cells after treatment in the study groups were washed with PBS, followed by 4% paraformaldehyde fixation. After rinsing, the cells were incubated with 3% Triton X-100 at room temperature for five minutes followed by a rinse with PBS. Then, the cells were incubated in the PBS containing 0.3% H_2_O_2_ for twenty minutes. Then, the cells were reacted with biotin-labeled solution which is prepared as described in the instructions for one hour at 37°C. Subsequently, streptavidin-HRP working solution was added into the cells. After diaminobenzidine staining, hematoxylin counterstain, the cell apoptosis was analyzed under a microscope (Olympus, Tokyo, Japan). Experiments were triplicated.

### 2.8. Western Blot

The extraction of the total proteins was performed in lysis buffer (50 mM DTT, 0.1% SDS and 1% NP-40) followed by centrifugation at 4°C (10,000 × *g*, 15 min). The supernatants were collected and protein was quantified using bicinchoninic acid (BCA) protein assay kit (Abcam, Cambridge, MA, USA). Then, electrophoresis of proteins (25 *µ*g) was performed on 15% SDS-PAGE followed by transferring to polyvinylidene fluoride membranes (Millipore, Billerica, MA, USA). After 5% skimmed milk blockage, the membranes were reacted with the primary antibodies against CNBP (1 : 100, cat no. ab272676, Abcam, Cambridge, MA, USA) overnight and then incubated with goat anti-rabbit secondary antibody (1 : 5000, cat no. ab216773, Abcam, Cambridge, MA, USA). An Odyssey infrared scanner (Li-Cor) was used for detection of the blots. Experiments were triplicated.

### 2.9. RNA Pull-Down Assay

The interaction between circFMN2 and CNBP was explored using RNA pull-down kits (Guangzhou Saicheng Biological Technology Co. LTD., Guangzhou, China) according to the instructions of manufacturer. Briefly, the cell lysates were prepared using lysis buffer followed by centrifugation. The probers including biotin-labeled circFMN2, biotin-labeled anti-sense circFMN2, and biotin-labeled circFMN2 fragments were incubated with streptomycin magnetic beads for six hours. Then, the magnetic bead-prober complex was obtained and incubated with the cell lysates overnight. The target protein was eluted and detected via Western blot assay.

### 2.10. RNA Immunoprecipitation

The interaction of circFMN2 and CNBP was further examined through the RNA immunoprecipitation assay using Imprint® RNA Immunoprecipitation (RIP) Kit (Sigma-Aldrich, St. Louis, MO, USA) in line with the protocol of manufacturer. Briefly, after cell lysis, the supernatants were collected and incubated with a magnetic bead anti-CNBP antibody complex or magnetic bead-IgG complex, respectively. After purification of the immunoprecipitated RNA, the RNA level was quantitatively analyzed via the RT-PCR assay.

### 2.11. Ubiquitination Assay

The cells were transfected with pcDNA3.1-CircFMN2 vector or its negative control and 5 *μ*mol/ml MG132 was added. Forty-eight hours after transfection, the cells were lysed and the supernatant was collected. Then, immunoprecipitation was performed via using anti-CNBP antibody and IgG. The immunoprecipitated protein was analyzed through Western blot assay via using anti-ubiquitin antibody (Cell Signaling Technology, Danvers, MA, USA).

### 2.12. Statistical Analysis

Statistical Product and Service Solutions (SPSS) 19.0 (IBM, Armonk, NY, USA) was used for data analysis. Difference between two groups was assessed using independent *t* tests. A 2-sided *p*-value under 0.05 suggested a significant difference.

## 3. Results

### 3.1. CircFMN2 Was Highly Expressed in Multidrug Resistance (MDR) Cells

**Figure 1:**
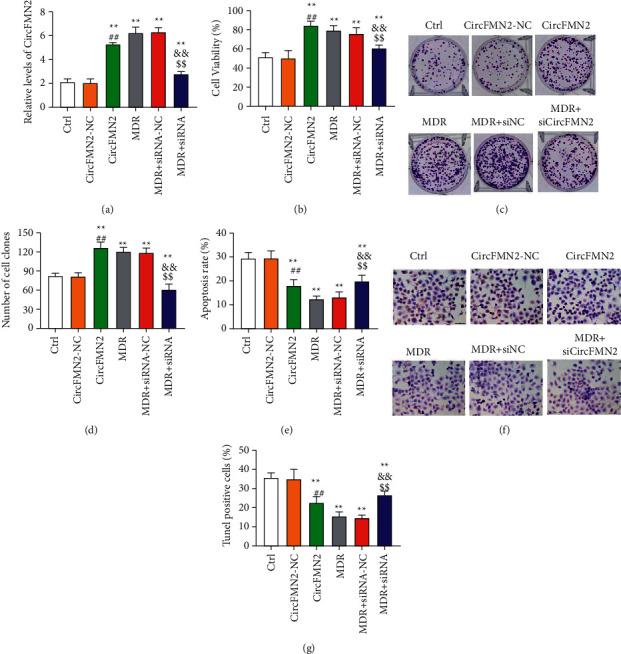
CircFMN2 was upregulated in MDR cells. The influence of circFMN2 on cell proliferation and apoptosis in multidrug-resistant cells and nonresistant cells. The level of circFMN2 assessed via RT-qPCR (a), cell proliferation measured by CCK-8 assay (b), colony formation ability (c) and (d), and cell apoptosis analyzed by flow cytometry (e) and TUNEL assay (f), (g) in the study groups. ^*∗∗∗*^*p* < 0.001, ^*∗∗*^*p* < 0.01 vs. control group; ^##^, *p* < 0.01, ^###^, *p* < 0.001 vs. circFMN2-NC group; ^&^*p* < 0.05, ^&&^*p* < 0.01, ^&&&^*p* < 0.001 vs. MDR group; ^$^, *p* < 0.05, ^$$^, *p* < 0.01, ^$$$^, *p* < 0.001 vs. MDR + siRNA-NC group.

In order to examine the underlying role of circFMN2 in BEL-7402 and BEL-7402/5-Fu cells, BEL-7402 and BEL-7402/5-Fu cells were transfected with pcDNA3.1-CircFMN2 vector and si-circFMN2, respectively. As revealed by the results of the PCR assay, the level of circFMN2 was elevated after circFMN2 transfection and decreased by si-circFMN2 transfection in comparison with MDR, suggesting that the overexpression and silencing of circFMN2 were successfully realized (Figure 1(a)). Besides, a significant increase of circFMN2 level was found in MDR group versus control, demonstrating that circFMN2 may act as a crucial player in multidrug-resistant cells. We further examined the effects of circFMN2 on cell proliferation and apoptosis after sorafenib treatment. As shown by the CCK-8 assay (Figure 1(b)) and colony formation assay (Figures 1(c) and 1(d)), circFMN2 overexpression significantly elevated cell viability and colony formation in BEL-7402 cells after sorafenib treatment, verifying the effect of circFMN2 overexpression on sorafenib resistance. Moreover, silencing of circFMN2 decreased cell proliferation in sorafenib treated MDR cells, indicating that knockdown of circFMN2 may be an efficient avenue in improving sorafenib resistance. The impact of circFMN2 on cell apoptosis after sorafenib treatment was assessed by flow cytometry and TUNEL assay. The apoptotic cells indicated by flow cytometry (Figure 1(e)) and TUNEL assay (Figures 1(f) and 1(g)), were increased significantly in sorafenib-treated MDR cells by circFMN2 depletion, further disclosed the effects of circFMN2 depletion on sorafenib resistance in MDR cells. On the other hand, this also indicated that circFMN2 is a crucial sorafenib resistant target in HCC.

### 3.2. CircFMN2 Elevated the CNBP Level via Restraining Its Ubiquitination Degradation

**Figure 2:**
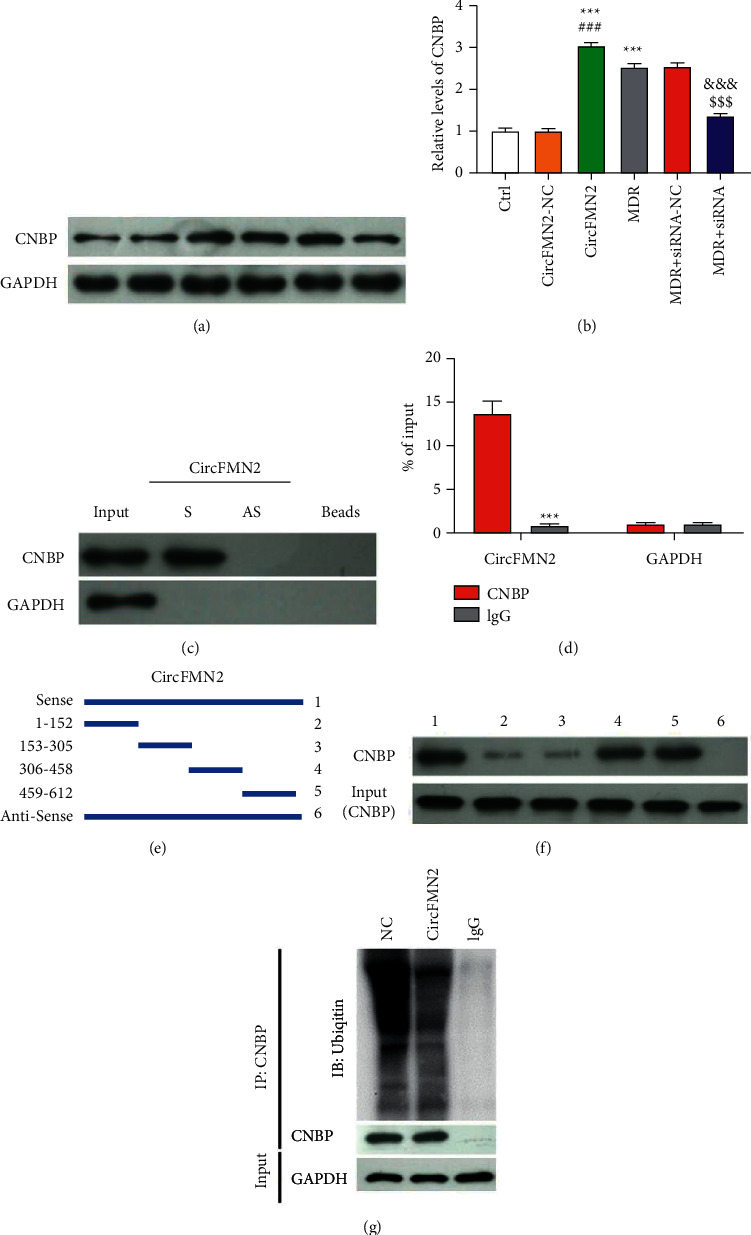
CNBP was confirmed as the binding protein of circFMN2. CNBP level was measured via Western blot (a), (b) lanes in panel A were corresponding to columns in panel B, Western blot analysis of CNBP level after RNA pull-down assay (c), circFMN2 level detected by RT-qPCR following RNA immunoprecipitation assay (d), The deletion fragments, sense strand and anti-sense strand of circFMN2 in deletion-mapping analysis (e), Western analysis of CNBP level following RNA pull-down assay with different circFMN2 constructs in deletion-mapping analysis (f), and ubiquitination level detected by Western blot assay (g). ^*∗∗∗*^*p* < 0.001 vs. control group; ^###^, *p* < 0.001 vs. circFMN2-NC group; ^&&&^, *p* < 0.001 vs. MDR group; ^$$$^, *p* < 0.001 vs. MDR + siRNA-NC group.

The downstream mechanism of circFMN2 in sorafenib resistance was further examined. CNBP was predicted as the binding protein of circFMN2 using bioinformatics online tools (StarBase and RNA interactome Database website). The CNBP level was measured via the Western blot assay. CNBP was upregulated by circFMN2 overexpression in BEL-7402 cells in contrast to control (Figures 2(a) and 2(b)), revealing that circFMN2 was an upregulator of CNBP. The level of CNBP was higher in MDR cells than that in BEL-7402 cells, suggesting that CNBP may be another drug resistance factor in MDR cells. The interaction between circFMN2 and CNBP was further validated through RNA-pull down and RNA immunoprecipitation assay, which revealed that the enrichment of CNBP was found in the circFMN2 with positive-sense strand group (Figure 2(c)). Moreover, it was observed that circFMN2 level was enriched in the CNBP immunoprecipitation group, and in contrast, very small amounts of circFMN2 were found in other groups (Figure 2(d)). These findings substantiated the binding of circFMN2 and CNBP. The binding sites of circFMN2 were further explored via deletion-mapping analysis and the results manifested that the CNBP was pulled down by circFMN2 fragments 306–458 nt and 459–612 nt (Figures 2(e) and 2(f)). The regulatory mechanism of circFMN2 on CNBP was also examined via ubiquitination assay. The ubiquitination level of CNBP was decreased transparently by the circFMN2 overexpression (Figure 2(g)), suggesting that circFMN2 overexpression elevates CNBP level via inhibiting its ubiquitination.

### 3.3. CircFMN2 Boosts Sorafenib Resistance in HCC Cells by Upregulating CNBP

**Figure 3:**
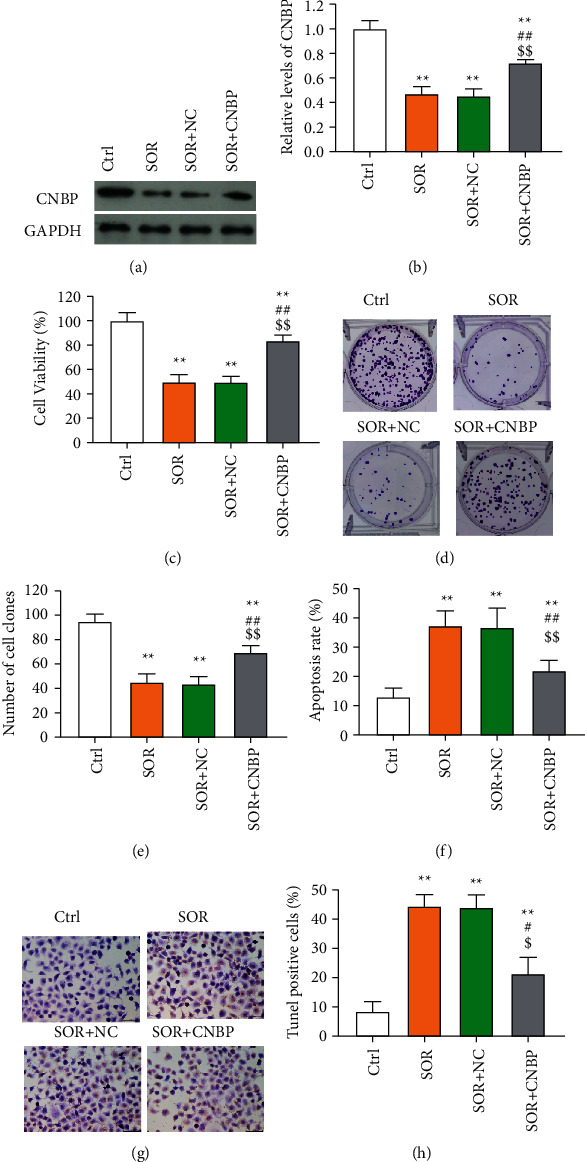
CNBP was upregulated in MDR cells. The influence of CNBP on cell proliferation and apoptosis in HCC cells. The level of CNBP assessed via Western blot (a) and (b), cell proliferation measured by CCK-8 assay (c); colony formation ability (d) and (e), and cell apoptosis analyzed by flow cytometry (f) and TUNEL assay (g), (h) in the study groups. ^*∗∗∗*^*p* < 0.001 vs. control group; ^#^, *p* < 0.05, ^##^, *p* < 0.01 and ^###^, *p* < 0.001 vs. SOR group; ^$^, *p* < 0.05, ^$$^, *p* < 0.01 and ^$$$^, *p* < 0.001 vs. SOR + NC group.

As stated in the aforementioned results, CNBP was upregulated in drug-resistant cells and may exert an underlying role in drug resistance, we therefore further examined the effects of CNBP on sorafenib efficacy in HCC cells. We found that CNBP was significantly decreased by sorafenib treatment in HCC cells when compared with control cells, and CNBP overexpression reversed this effect (Figures 3(a) and 3(b)). The cell viability and colony formation capacity were all reduced significantly by sorafenib in HCC cells (Figures 3(c)–3(e)). Moreover, the cell apoptosis assessed by flow cytometry and positive cells in TUNEL staining were all increased by sorafenib in HCC cells (Figures 3(f)–3(h)). These results were consistent with the previous findings [24, 25], demonstrating an antiproliferation and proapoptosis role of sorafenib in HCC. CNBP was high expressed in MDR cells and after CNBP overexpression, the effects of sorafenib on cell apoptosis and proliferation in HCC cells were reversed, supporting that CNBP acts as a contributor in sorafenib resistance. Besides, it has been confirmed in the aforementioned results that upregulation of CNBP was realized via inhibition of ubiquitination by circFMN2.

## 4. Discussion

Sorafenib as a first-line anticancer drug, has become a standard treatment of advanced liver cancer, wining a crucial position in HCC therapy [26–28]. The sorafenib therapy has yielded a modest survival benefit in patients with advanced HCC [29, 30]. Nevertheless, sorafenib treatment still confronts tremendous challenges in sorafenib resistance [31]. The sorafenib-resistant mechanism is still not completely unambiguous and its elucidation is imperative. In the present report, we found that circFMN2 facilitates sorafenib resistance via upregulating CNBP through restraining inhibiting ubiquitination.

As revealed by accumulative evidence, aberrant expressed noncoding RNAs has become an essential factor in HCC therapy and sorafenib resistance [32, 33]. A lot of research on treatment of liver cancer and sorafenib resistance are focused on aberrantly expressed miRNA or lncRNA [33–38]. Nevertheless, the reports of circRNA participating in sorafenib resistance are limited. Compared with miRNAs and lncRNAs, circRNAs, owing to its stable circular structure, has more potential to be an excellent treatment target and drug resistant biomarker. In the present study, circFMN2, a newly found circRNA in cancerous cells, was found to be upregulated in MDR HCC cells, implying its underlying role in drug resistance.

Cell proliferation and apoptosis are critical indicators of drug resistance. *β*-catenin regulated by Nek2 contributes to sorafenib resistance via regulating cell apoptosis and proliferation [39]. Rage participates in sorafenib resistance via modulating proliferation and apoptosis by the AMPK/mTOR pathway [40]. CircFMN2 was reported to exert a carcinogenic effect in colorectal cancer and prostate cancer cells via regulating cell proliferation and apoptosis [22, 23]. In this research study, we found that after circFMN2 overexpression, the cell viability and colony formation capacity were increased and cell apoptosis was reduced in sorafenib treated cells, hinting that circFMN2 is a critical functional molecular in facilitating sorafenib resistance. As circFMN2 is highly expressed in MDR cells, we further conducted experiments into the effects of circFMN2 depletion on cell apoptosis and proliferation in sorafenib treated MDR cells. Surprisingly, circFMN2 depletion displayed mitigative effects on sorafenib resistance via augmenting cell apoptosis and suppressing cell proliferation in multidrug resistance cells. The outcome disclosed that circFMN2 is an underlying sorafenib resistance target; on the other hand, high level of circFMN2 is the crucial inducer of sorafenib resistance.

CircRNAs as critical regulators, commonly work by regulating their downstream miRNA targets, binding proteins, and certain signaling pathways. As reported in the previous literature, circRNA-SORE is found to facilitate sorafenib resistance through *β*-catenin signaling in liver cancer [41]. CircFN1 augments sorafenib resistance via sponging miR-1205 and modulating the expression of e2f1 in HCC cells [42]. In the present study, we sought to find more drug resistant by exploring the downstream mechanism of circFMN2.

As pinpointed by the bioinformatics tools, CNBP was predicted as the binding protein of circFMN2. CNBP is ubiquitous in various tissues and organs, exerting dual regulator functions at translational and transcriptional levels [43, 44]. In this report, CNBP has been identified as the binding protein via RNA pull-down and RNA immunoprecipitation assay. As further verified by deletion-mapping analysis, CNBP is capable to bind to circFMN2 fragments 306–458 nt and 459–612 nt. Besides, circFMN2 upregulated CNBP by ubiquitination inhibition. Since circFMN2 is an underlying MDR target, we speculated that CNBP as a downstream binding protein may have similar effects. We found that CNBP is also upregulated in MDR cells versus nonresistant cells, which confirmed our speculation. Many studies have shown that CNBP is a vital regulator of cell apoptosis and proliferation [45–47]. Besides, CNBP is also confirmed to act as a crucial regulator of cell biology by modulating oncogene expression in tumor [48].

## 5. Conclusions

In this research, circFMN2, a drug resistant target was found in HCC cells. We also identified a new sorafenib-resistant mechanism that circFMN2 contributes to sorafenib resistance via upregulation of CNBP through ubiquitination inhibition. The findings of this research provide a new solution for sorafenib resistance and extend our interest in sorafenib resistance in HCC.

## Data Availability

The data used to support the findings of this study are available from the corresponding author upon request.
